# Thyroid-Stimulating Hormone and Free Thyroxine Levels at Labor Admission: Associations with Obstetric and Neonatal Outcomes in Term Pregnancies

**DOI:** 10.3390/diagnostics16040595

**Published:** 2026-02-17

**Authors:** Karolin Ohanoglu Cetinel, Yıldız Karademir, Turan Arda Demirag, Bugra Tunc, Osman Murat Guler, Alperen İnce

**Affiliations:** 1 Department of Obstetrics and Gynecology, Basaksehir Cam and Sakura City Hospital, 34480 Istanbul, Türkiye; dryildizkarademir@gmail.com (Y.K.);; 2 Department of Obstetrics and Gynecology, Mersin Aydincik State Hospital, 33840 Mersin, Türkiye; dr.bgrtnc@gmail.com; 3 Department of Obstetrics and Gynecology, Tekirdag Kapakli State Hospital, 59510 Tekirdag, Türkiye; omguler12@gmail.com; 4 Department of Obstetrics and Gynecology, Konya Bozkir State Hospital, 42630 Konya, Türkiye; alperenince@gmail.com

**Keywords:** birth weight, free thyroxine, maternal thyroid function, labor admission, term pregnancy

## Abstract

**Background:** Maternal thyroid hormones are essential for fetal development and the maintenance of pregnancy. While thyroid dysfunction earlier in gestation has been extensively studied, the clinical relevance of thyroid function assessed at labor admission remains unclear. This study investigated the association between maternal thyroid function parameters measured at labor ward admission and obstetric and neonatal outcomes in term pregnancies. **Methods:** In this retrospective observational study, 664 women with singleton term pregnancies (≥37 weeks) admitted to the labor ward of a tertiary referral center were included. Maternal thyroid-stimulating hormone (TSH), free thyroxine (FT4), and admission complete blood count parameters (hemoglobin, hematocrit, white blood cell count, and platelet count) were recorded. Obstetric and neonatal outcomes were compared across FT4 tertiles using univariable and multivariable regression analyses adjusted for key obstetric confounders. **Results:** Gestational age at delivery differed significantly across FT4 tertiles, with higher FT4 levels associated with a greater proportion of late-term deliveries. Lower FT4 levels were independently associated with lower neonatal birth weight categories after adjustment for gestational age and parity. Admission complete blood count parameters did not differ significantly across FT4 tertiles or gestational age categories. Maternal TSH levels were not independently associated with obstetric or neonatal outcomes, and no significant associations were observed with Apgar scores or NICU admission. **Conclusions:** In term pregnancies, maternal FT4 levels measured at labor admission are associated with delivery timing and neonatal birth weight but do not independently predict intrapartum fetal distress or adverse immediate neonatal outcomes.

## 1. Introduction

Thyroid hormones are crucial for maternal metabolic regulation and fetal development throughout pregnancy. Adequate maternal thyroid function is vital for maintaining placental function, ensuring appropriate uteroplacental perfusion, and supporting fetal neurodevelopment, especially in the last weeks of gestation [1,2,3]. Importantly, even small changes in levels of thyroid-stimulating hormone (TSH) and free thyroxine (FT4) within the reference range have been associated with adverse pregnancy outcomes in observational studies [4,5,6].

Previous research has suggested that maternal thyroid dysfunction may influence the duration of pregnancy, mode of delivery, and perinatal outcomes such as birth weight, Apgar scores, and neonatal intensive care unit (NICU) admission [7,8,9]. Elevated maternal TSH concentrations have been associated with an increased risk of cesarean delivery and intrapartum fetal compromise, while isolated alterations in FT4 levels have been linked to impaired fetal growth and suboptimal neonatal adaptation [10,11,12]. However, the findings differ significantly in terms of the outcomes examined and the timing of thyroid function assessment.

Most studies that examined maternal thyroid function levels have focused on antenatal measurements conducted during early or mid-pregnancy. However, data regarding thyroid hormone levels at the time of admission for labor are limited [13]. The intrapartum period is characterized by profound endocrine, inflammatory, and hematologic changes that may influence both maternal and neonatal outcomes [14,15,16]. In addition, there are limited studies regarding the possible link between maternal thyroid function at admission and postpartum hematologic changes.

While thyroid hormone level assessments conducted earlier in pregnancy are informative about long-term obstetric risk, they may not necessarily indicate the endocrine profile immediately prior to delivery. Accordingly, thyroid function tests performed at the time of labor may better reflect late gestational thyroid physiology than antenatal measurements, which may be influenced by transient hormonal fluctuations.

Despite this potential relevance, evidence regarding the clinical significance of thyroid hormone levels measured at labor admission remains scarce. Available studies often limited by small sample sizes, selective populations, or a primary focus on overt dysfunction, leaving the impact of subtle hormonal variations within reference insufficiently characterized. Therefore, the present retrospective study aimed to evaluate the association between maternal TSH and FT4 levels measured at labor ward admission and (i) gestational age at delivery, (ii) mode of delivery, (iii) early postpartum complete blood count (CBC) changes, and (iv) neonatal outcomes, including Apgar scores, birth weight, and NICU admission, in a large cohort of term pregnancies.

## 2. Materials and Methods

### 2.1. Study Design and Setting

This retrospective observational study was conducted at the labor ward of Basaksehir Cam ve Sakura City Hospital, Istanbul, Türkiye. Medical records of pregnant women admitted for delivery between 30 May 2020 and 30 May 2025 were reviewed. This study was carried out in accordance with the principles of the Declaration of Helsinki, and the study protocol was approved by the Institutional Ethics Committee of Basaksehir Cam and Sakura City Hospital, Istanbul, Türkiye. Since it was a retrospective study, informed consent was not required.

### 2.2. Study Population

Eligible participants were pregnant women with singleton pregnancies who were admitted to the labor ward with an initial plan for normal vaginal delivery at ≥37 gestational weeks. Gestational age was determined based on first-trimester ultrasonography using crown–rump length measurements obtained between 8 and 14 weeks of gestation, in accordance with institutional dating criteria.

Women who delivered vaginally as well as those who required intrapartum cesarean delivery, including cesarean delivery due to fetal distress, after admission were included in the analysis to allow evaluation of delivery outcomes among women initially admitted for vaginal birth management.

To minimize potential confounding, women with coexisting medical conditions—including chronic hypertension, preeclampsia, diabetes mellitus, gestational diabetes mellitus, or other chronic systemic diseases—were excluded. Additionally, women who required perinatology unit follow-up, those with multiple gestations or major fetal anomalies, and those with incomplete clinical or laboratory data were not included in the analysis.

During the study period, medical records of approximately 15,000 pregnant women who delivered at our institution were reviewed. A total of 664 patients who met the predefined inclusion and exclusion criteria were ultimately included in the study.

### 2.3. Data Collection and Obstetric Outcomes

Demographic and obstetric characteristics, including maternal age and parity, were extracted from electronic medical records. Gestational age at delivery was recorded and analyzed both as a continuous variable and categorically (e.g., 37, 38, 39, 40, and 41–42 weeks), where appropriate.

Mode of delivery was categorized as normal vaginal delivery, cesarean delivery due to fetal distress, or cesarean delivery due to indications other than fetal distress. At our institution, only women with a clear indication for planned vaginal delivery are admitted to the labor ward. Pregnant women with absolute indications for cesarean delivery, such as malpresentation or contraindications to vaginal birth, are scheduled directly for elective cesarean delivery and are not admitted to the labor ward.

During labor follow-up, cesarean delivery was performed only if specific intrapartum indications developed after admission for planned vaginal birth. In our study, cesarean delivery for indications other than fetal distress includes intrapartum cesarean sections undertaken for the management of disorders of labor arrest, for example, arrest of cervical dilation and arrest of fetal descent, and for the management of suspected cephalopelvic disproportion that develops during active labor. Active labor was defined as cervical dilation of ≥4 cm in the presence of effective uterine contractions.

### 2.4. Laboratory Measurements

Maternal thyroid function tests, including thyroid-stimulating hormone (TSH) and free thyroxine (FT4), were obtained for all participants at the time of admission to the labor ward, prior to delivery. Third-trimester reference ranges were used for interpretation (TSH: 0.41–5.18 μIU/mL; FT4: 0.7–1.20 ng/dL).

Admission complete blood count (CBC) parameters, including hemoglobin (Hb), hematocrit (Hct), white blood cell count (WBC), and platelet count (PLT), were recorded at the same time point. Postpartum CBC measurements were routinely performed at the 6th hour after delivery or cesarean section. Changes in hematological parameters were calculated as the difference between postpartum 6 h values and admission values (ΔHb, ΔHct).

### 2.5. Neonatal Outcomes

Neonatal outcomes included birth weight, Apgar scores at both 1 and 5 min, and admission to the neonatal intensive care unit (NICU). Admission to the NICU was recorded as a binary variable (yes/no) based on clinical indications set by the attending neonatologist. Due to the diversity in the indications for admission to the NICU and the low incidence in term pregnancy, admission to the NICU was used as an exploratory short-term neonatal outcome.

### 2.6. Statistical Analysis

Statistical analyses were performed using IBM SPSS Statistics for Windows, version 29.0 (IBM Corp., Armonk, NY, USA). The distribution of continuous variables was assessed using the Shapiro–Wilk test. Continuous variables with non-normal distribution are presented as median (interquartile range), while categorical variables are presented as number and percentage.

Comparisons among gestational age categories and FT4 tertiles were performed using the Kruskal–Wallis test for continuous variables and the chi-square test or Fisher’s exact test for categorical variables, as appropriate. When overall comparisons were statistically significant, post hoc pairwise comparisons were conducted using the Mann–Whitney U test, with the term gestational age group or the middle FT4 tertile used as the respective reference categories. A two-sided *p* value < 0.05 was considered statistically significant.

The authors acknowledge the use of a generative artificial intelligence tool (ChatGPT 5.2, OpenAI, San Francisco, CA, USA) for language refinement and assistance with reference formatting during manuscript preparation.

## 3. Results

A total of 664 pregnant women were included in the study and categorized into early-term (37–38 weeks; *n* = 266), term (39–40 weeks; *n* = 312), and late-term (≥41 weeks; *n* = 86) groups (Table 1). The maternal ages were not significantly different among the gestational age groups (*p* = 0.051). Parity was significantly higher in the early-term group than in the term group (*p* < 0.001), whereas parity was comparable between the term and late-term groups (*p* = 0.597). The admission TSH levels were similar among the gestational age groups (*p* = 0.608). No statistically significant difference in FT4 levels was observed among gestational age groups (*p* = 0.053).

The admission hematological parameters, including hemoglobin, hematocrit, white blood cell count, and platelet count, were not significantly different among early-term, term, and late-term deliveries (*p* = 0.821 for hemoglobin, *p* = 0.948 for hematocrit, *p* = 0.270 for white blood cell count, and *p* = 0.580 for platelet count). The admission levels of hemoglobin, hematocrit, white blood cell count, and platelet count were similar among the FT4 tertile groups, and no significant differences were observed among the groups (*p* > 0.05 for all parameters). The changes in hemoglobin and hematocrit levels in the early postpartum period, ΔHb and ΔHct, were similar among the gestational age groups and FT4 tertile groups.

Mode of delivery differed significantly among gestational age categories (*p* < 0.001). Birth weight (g) also differed significantly across groups (*p* < 0.001), with lower birth weights observed in the early-term group and higher birth weights in the late-term group compared with the term group. No statistically significant differences were observed among groups with respect to 1 min Apgar scores (*p* = 0.589), 5 min Apgar scores (*p* = 0.628), or NICU admission rates (*p* = 0.078) (Table 2). NICU admission was evaluated as an exploratory outcome due to its low incidence and is therefore reported descriptively.

In stratified sensitivity analyses comparing early-term (37–38 weeks) and ≥39-week deliveries, the direction of the association between FT4 levels and neonatal birth weight remained consistent across strata, with no evidence of effect modification (Supplementary Table S1).

Among women who delivered by cesarean section, admission thyroid function parameters were comparable across gestational age categories. Median TSH levels were not significantly different in early-term, term, and late-term deliveries (*p* = 0.525). Similarly, median levels of FT4 were comparable across gestational age groups, with no statistically significant difference observed (*p* = 0.116) (Table 3).

No significant differences in 1 min or 5 min Apgar scores were observed across FT4 or TSH tertiles within any gestational age category (all *p* > 0.05); therefore, Apgar scores were not included in multivariable analyses.

Gestational age at delivery differed significantly across FT4 tertiles (*p* = 0.018), with a progressively higher proportion of late-term deliveries (≥41 weeks) observed in the higher FT4 tertiles (*p* = 0.031) (Table 4).

The frequency of cesarean delivery due to fetal distress differed significantly among FT4 tertiles, being highest in the lowest FT4 tertile and lowest in the highest FT4 tertile (*p* = 0.044). Birth weight increased significantly across FT4 tertiles (*p* < 0.001) (Table 4).

In multivariable logistic regression analysis, FT4 levels standardized per 1 SD decrease were not independently associated with cesarean delivery due to fetal distress after adjustment for gestational age and parity (*p* = 0.90). Higher parity remained independently associated with substantially lower odds of cesarean delivery due to fetal distress (adjusted OR 0.16, 95% CI 0.08–0.31; *p* < 0.001) (Table 5).

In the multivariable ordinal logistic regression analysis, lower FT4 levels at admission were independently associated as a predictor for lighter categories of newborn weight. This association persisted after adjusting for both gestational age and parity (adjusted OR per 1 SD decrease, 1.19; 95% CI: 1.02 to 1.38; *p* = 0.025). Conversely, positive associations were observed between longer gestational age and higher parity, predicting heavier categories of newborn weight (both results significant, *p* < 0.001 for gestational age and *p* = 0.001 for parity) (Table 6).

## 4. Discussion

In this retrospective cohort study of term pregnancies, maternal thyroid function parameters measured at labor ward admission demonstrated selective associations with delivery timing and neonatal birth weight. After adjustment for key obstetric confounders, no independent association was identified between admission thyroid hormone levels and cesarean delivery for fetal distress. These findings indicate that intrapartum thyroid hormone measurements should be interpreted within their physiological and obstetric context, rather than as isolated predictors of acute intrapartum complications.

The primary contribution of this study is its examination of maternal thyroid function at labor ward admission in otherwise uncomplicated term pregnancies. In contrast to most previous research, which has focused on thyroid hormone measurements from early or mid-pregnancy, this study addresses a late-gestational period that has received limited attention. By evaluating TSH and FT4 levels at admission in relation to gestational age at delivery, neonatal birth weight, and short-term neonatal outcomes, the findings provide insight into how subtle variations in thyroid hormones within reference ranges may be associated with outcomes near delivery. The study highlights physiological associations rather than predictive or diagnostic claims, offering a nuanced understanding of late-gestation thyroid physiology without overstating clinical implications.

Gestational age at delivery differed across FT4 tertiles, with higher FT4 levels linked to a greater proportion of late-term deliveries. This observation aligns with epidemiological evidence suggesting a relationship between maternal thyroid hormone availability and timing of parturition. Large-scale studies and meta-analyses, including those by Korevaar and colleagues, have shown associations between deviations in maternal thyroid hormone levels and altered risks of preterm or early-term birth [17]. These findings should be interpreted as descriptive, population-level associations rather than evidence for clinical prediction or screening. Although most previous studies assessed thyroid function earlier in pregnancy, our findings suggest that FT4 levels at labor admission may be associated with pregnancy duration. However, without longitudinal thyroid function data, this association should be seen as a potential marker of late-gestation thyroid physiology rather than evidence of cumulative endocrine exposure.

The physiological adaptation of the maternal thyroid axis during pregnancy is complex and dynamic, particularly in the period approaching term. During this time, placental transport mechanisms, deiodinase activity, and the increasing autonomy of the fetal thyroid gland collectively regulate fetal exposure to thyroxine [18,19,20]. Thyroid hormones are central to fetal growth and maturation through their regulatory effects on placental function, nutrient transport, and fetal anabolic signaling pathways [19,20]. In this context, it is plausible that small changes in maternal free thyroxine levels, even within the normal range, may be associated with differences in fetal growth and gestational age at delivery.

An inverse association between FT4 tertiles and the incidence of cesarean delivery due to fetal distress was observed in unadjusted analyses; however, this association was no longer evident after adjustment for gestational age and parity. This finding indicates that the initial association was largely confounded by established obstetric determinants of fetal tolerance during labor. Parity and gestational age, both well-recognized predictors of labor progression and fetal reserve, appeared to exert a stronger influence than biochemical parameters, indicating that maternal thyroid hormone levels at admission do not independently predict fetal distress in labor [16].

From a clinical perspective, particularly in high-volume tertiary labor wards, thyroid function tests performed at the time of hospital admission are often performed for practical or institutional rather than patient-specific prognostic purposes. Our results support the view that intrapartum thyroid hormone levels in pregnancy do not play a significant role in counseling patients or intrapartum decision-making for uncomplicated term pregnancies.

In contrast, lower FT4 levels at admission remained independently associated with lower neonatal birth weight categories after adjustment for gestational age and parity. Although this association was statistically significant, the observed effect size was modest. Therefore, these findings do not support a predictive or screening role for intrapartum FT4 measurements in clinical practice. Rather, the association might be explained by the influence of thyroid hormone supply on the fetal growth curve. FT4 levels at admission are therefore considered a contextual factor in the mother’s endocrine status towards the end of gestation, rather than a determinant of the neonate’s birth weight.

Maternal TSH levels at admission were not associated with gestational age, mode of delivery, or neonatal outcomes in our cohort. This is in accordance with previous findings suggesting that free thyroxine (FT4) levels could be considered a more biologically relevant indicator of available thyroid hormone in late pregnancy, whereas levels of TSH tend to remain relatively stable in the face of large variations in circulating levels of thyroxine [17,18]. During late pregnancy, the activity of placental deiodinases and the dynamics of thyroid hormone binding could also limit the relationship between TSH and peripheral tissue thyroid hormone activity.

We also observed no significant association between maternal thyroid function parameters at admission and immediate neonatal outcomes, including Apgar scores and NICU admission. These findings are consistent with recent intrapartum-focused evidence reported by Sivas et al., who showed that maternal thyroid hormone measurements obtained at labor admission were not independently associated with short-term neonatal morbidity in term pregnancies [21]. It is also noteworthy that NICU admission is a multifactorial outcome that is dependent on a wide variety of neonatal and perinatal factors, and as a result, this outcome must be viewed as an exploratory outcome for this particular study due to a low event rate for this particular outcome measure. The findings suggest that thyroid hormone fluctuations in labor are a physiological response rather than a response to acute compromise in uncomplicated term pregnancies.

Regarding hematologic parameters, no significant associations were observed between thyroid function at admission and postpartum changes in complete blood count values. The lack of significant associations indicates that thyroid hormone levels in the intrapartum period do not influence hematologic parameters in the postpartum period in low-risk term pregnancies.

### 4.1. Strengths and Limitations

The strengths of this study include a well-defined cohort restricted to term pregnancies, standardized measurement of thyroid function parameters at the time of labor ward admission, and the use of multivariable regression analyses to adjust for key obstetric confounders. These design features enhance the internal validity of the findings and allow for a focused evaluation of intrapartum thyroid function.

Several limitations should also be acknowledged. The retrospective study design precludes causal inference. Thyroid hormone measurements were obtained at a single intrapartum time point, and data on thyroid autoantibody status were not available. In addition, although the overall sample size was substantial, the number of cesarean deliveries due to fetal distress was relatively limited, which may have reduced the statistical power for this specific outcome.

### 4.2. Clinical Implications

From a clinical perspective, the present findings suggest that routine assessment of TSH or FT4 at labor admission should not be used in isolation to predict intrapartum fetal distress or immediate neonatal morbidity in uncomplicated term pregnancies. The lack of an independent association with cesarean delivery due to fetal distress after multivariable adjustment underscores the importance of established obstetric factors over biochemical parameters in intrapartum risk assessment.

However, the observed independent association between lower FT4 levels and reduced neonatal birth weight indicates that maternal thyroid hormone status may still be relevant to fetal growth trajectories, even in late gestation. This association likely reflects longer-term influences of maternal thyroid hormone availability rather than acute intrapartum effects.

## 5. Conclusions

In conclusion, maternal FT4 levels measured at admission to the labor ward are associated with gestational age at delivery and neonatal birth weight but do not independently predict cesarean delivery due to fetal distress or adverse immediate neonatal outcomes. These findings highlight the complex and context-dependent role of thyroid hormones in late pregnancy and support a cautious interpretation of intrapartum thyroid function tests in clinical practice.

## Figures and Tables

**Table 1:** Baseline maternal characteristics and admission laboratory parameters according to gestational age category.

Variable	Early Term (37–38 Weeks)	Term (39–40 Weeks)	Late Term (≥41 Weeks)	*p* Value
Maternal age (years)	25.0 (22.0–28.0)	24.0 (22.0–27.0)	24.0 (22.0–27.8)	0.051
Parity	2.0 (1.0–2.8)	1.0 (1.0–2.0)	1.0 (1.0–2.0)	<0.001
TSH (μIU/mL)	1.62 (1.13–2.34)	1.74 (1.11–2.44)	1.77 (1.02–2.67)	0.608
FT4 (ng/dL)	1.01 (0.88–1.16)	0.97 (0.86–1.10)	0.99 (0.90–1.12)	0.053
Hemoglobin at admission (g/dL)	11.4 (10.6–12.4)	11.5 (10.5–12.5)	11.6 (10.5–12.6)	0.821
Hematocrit at admission (%)	34.7 (32.6–37.2)	35.0 (32.2–37.2)	35.2 (32.2–38.0)	0.948
White blood cell count (×10^9^/L)	10.4 (9.0–12.0)	10.4 (9.1–12.3)	10.1 (9.1–11.8)	0.270
Platelet count (×10^9^/L)	237 (198–287)	245 (202–282)	255 (201–293)	0.580

Values are presented as median (interquartile range). Comparisons among gestational age groups were performed using the Kruskal–Wallis test.

**Table 2:** Obstetric and neonatal outcomes according to gestational age category.

Outcome	Early Term (37–38 Weeks; n = 266)	Term (39–40 Weeks; n = 312)	Late Term (≥41 Weeks; n = 86)	*p* Value
Mode of delivery, n (%)				<0.001
Normal vaginal delivery	77 (28.9)	189 (60.6)	47 (54.7)	
Cesarean delivery due to fetal distress	30 (11.3)	31 (9.9)	12 (14.0)	
Cesarean delivery due to other indications	159 (59.8)	92 (29.5)	27 (31.4)	
Birth weight (grams)	3070 (2710–3370)	3330 (3060–3630)	3680 (3300–4080)	<0.001
Apgar score at 1 min	8.0 (7.0–8.0)	8.0 (7.8–8.0)	8.0 (7.0–8.0)	0.589
Apgar score at 5 min	9.0 (9.0–9.0)	9.0 (9.0–9.0)	9.0 (9.0–9.0)	0.628
NICU admission, n (%)	10 (3.8)	3 (1.0)	2 (2.3)	0.078

Continuous variables are presented as median (interquartile range), and categorical variables are presented as number and percentage. The Kruskal–Wallis test was used for continuous variables, while the chi-square test or Fisher’s exact test was used for categorical variables, as appropriate.

**Table 3:** Analysis of admission TSH and FT4 values among women who delivered by cesarean section according to gestational age category.

Parameter	Group	n	Median (IQR)	*p* Value
TSH (μIU/mL)	Early term (37–38 w)	178	1.62 (1.18–2.42)	0.525
	Term (39–40 w)	120	1.75 (1.23–2.51)	
	Late term (≥41 w)	37	1.93 (1.02–2.76)	
FT4 (ng/dL)	Early term (37–38 w)	179	0.99 (0.87–1.15)	0.116
	Term (39–40 w)	116	0.95 (0.86–1.07)	
	Late term (≥41 w)	37	1.02 (0.88–1.12)	

Values are presented as median (interquartile range). Comparisons among gestational age categories were performed using the Kruskal–Wallis test. Abbreviations: w: week

**Table 4:** Obstetric and neonatal outcomes according to FT4 tertiles at admission.

Outcome	FT4 T1 (Low) n = 219	FT4 T2 (Mid) n = 218	FT4 T3 (High) n = 219	*p* Value
Gestational age at delivery (weeks)	39.0(38.0–40.0)	39.0 (38.0–40.0)	40.0 (39.0–41.0)	0.018
Early-term delivery (37–38 w), n (%)	72 (32.9)	66 (30.3)	54 (24.7)	0.031
Term delivery (39–40 w), n (%)	104 (47.5)	108 (49.5)	101 (46.1)	
Late-term delivery (≥41 w), n (%)	43 (19.6)	44 (20.2)	64 (29.2)	
Cesarean delivery due to fetal distress, n (%)	32 (14.6)	25 (11.5)	16 (7.3)	0.044
Birth weight (grams)	3120 (2850–3420)	3290 (3010–3580)	3510 (3180–3860)	<0.001

Data are presented as median (interquartile range) or number (percentage), as appropriate. Comparisons among FT4 tertiles were performed using the Kruskal–Wallis test for continuous variables and the chi-square test for categorical variables. FT4 tertiles were defined based on the distribution of admission FT4 values. A *p* value < 0.05 was considered statistically significant.

**Table 5:** Multivariable logistic regression analysis for cesarean delivery due to fetal distress.

Variable	Adjusted OR	95% CI	*p* Value
FT4 (per 1 SD decrease)	1.01	0.88–1.16	0.90
Gestational age (per 1-week increase)	0.91	0.74–1.13	0.41
Parity (per 1-unit increase)	0.16	0.08–0.31	<0.001

Outcome: cesarean delivery due to fetal distress (yes/no). Predictors: FT4 (standardized), gestational age, and parity (N = 637; events = 66). Adjusted odds ratios were obtained from multivariable logistic regression. FT4 was standardized (z-score; 1 SD ≈ 0.20 ng/dL); values outside the clinically plausible range (0.4–2.5 ng/dL) were excluded.

**Table 6:** Multivariable ordinal logistic regression analysis for birth weight categories.

Variable	Adjusted OR *	95% CI	*p* Value
FT4 (per 1 SD decrease)	1.19	1.02–1.38	0.025
Gestational age (per 1-week increase)	1.91	1.68–2.18	<0.001
Parity (per 1-unit increase)	1.34	1.13–1.59	0.001

Outcome: birth weight category (<3000 g, 3000–3500 g, 3500–4000 g, >4000 g). Predictors: FT4 (standardized), gestational age, and parity (N = 620). * Adjusted odds ratios were obtained from multivariable ordinal logistic regression. FT4 was standardized (z-score; 1 SD ≈ 0.20 ng/dL).

## Data Availability

Dataset available on request from the authors. The raw data supporting the conclusions of this article will be made available by the authors on request.

## Supplementary Materials

The following supporting information can be downloaded at: https://www.mdpi.com/article/10.3390/diagnostics16040595/s1, Table S1: Stratified multivariable analyses of the association between FT4 levels and neonatal birth weight according to gestational age category.
