# Supplementary material for: Thyroid-Stimulating Hormone and Free Thyroxine Levels at Labor Admission: Associations with Obstetric and Neonatal Outcomes in Term Pregnancies
Source: Diagnostics (Basel). 2026 Feb 17;16(4):595. doi: 10.3390/diagnostics16040595 (PMC12939775; doi:10.3390/diagnostics16040595)
Supplement: Supplementary file 1 [file diagnostics-16-00595-s001.zip › diagnostics-4146531-supplementary.pdf]

Supplementary Table S1

Stratified multivariable analyses of the association between FT4 levels and neonatal birth weight according to gestational age category.

Gestational age category	FT4 coefficient (β)	95% CI (lower)	95% CI (upper)	p value
37–38 weeks	-0.0002	-0.0006	0.0003	0.4962
≥39 weeks	-0.1884	-0.8335	0.4567	0.5661

Models adjusted for gestational age at delivery and parity. No significant interaction between FT4 levels and gestational age category was observed.
